# A tool to define and measure maternal healthcare acceptability at a selected health sub-district in South Africa

**DOI:** 10.1186/s12884-023-05475-y

**Published:** 2023-04-29

**Authors:** Joy Blaise Bucyibaruta, Mmapheko Doriccah Peu, Lesley Bamford, Alfred Musekiwa

**Affiliations:** 1 School of Health Systems and Public Health, Faculty of Health Sciences, University of Pretoria, Pretoria, South Africa; 2 Department of Nursing Sciences, Faculty of Health Sciences, University of Pretoria, Pretoria, South Africa; 3 National Department of Health, Pretoria, South Africa

**Keywords:** Access to healthcare, Community support, Definition, Delphi technique, Healthcare stakeholders, Healthcare systems, Maternal healthcare acceptability, Measurement tool, Public health, Social psychology

## Abstract

**Background:**

There are many factors during pregnancy and labor that influence women’s acceptability of maternal healthcare. Nevertheless, the concept of acceptability of maternal healthcare has unfortunately not been clearly defined and remains difficult to assess, affecting its implications and approaches from maternal health perspectives. In this study, we proposed a practical definition of maternal healthcare acceptability and developed a tool to measure maternal healthcare acceptability from patients’ perspective at a selected health sub-district in South Africa.

**Methods:**

We applied known techniques to develop measurement tools in health settings. The concept development drew from the literature review leading to the proposed definition of maternal healthcare acceptability which was then refined and validated by experts through Delphi technique. Other techniques included specification of concept constructs; selection of indicators; formation of indices; measurement tool/scale construction; and testing of reliability and validity. Factor analysis and simple arithmetic equation were performed on secondary and primary datasets respectively.

**Results:**

Experts in the field reached a consensual definition of maternal healthcare acceptability. Factor analysis revealed three factors retained to predict maternal healthcare acceptability indices, namely provider, healthcare and community. Structural equation model showed good fit (CFI = 0.97), with good reliability and validity. Hypothesis testing confirmed that items and their corresponding factors were related (*p* < 0.01). Simple arithmetic equation was recommended as alternative method to measure acceptability when factor analysis was not applicable.

**Conclusion:**

This study provides new insights into defining and measuring acceptability of maternal healthcare with significant contributions on existing theories and practices on this topic and practical applications not only for maternal health but also across diverse health disciplines.

**Supplementary Information:**

The online version contains supplementary material available at 10.1186/s12884-023-05475-y.

## Introduction

Acceptability of healthcare is an emerging concept which is rapidly evolving to become essential in planning, implementing and assessing healthcare services [1, 2]. Healthcare acceptability can be applied to a wide range of healthcare services [3, 4]. For example, women attending antenatal, delivery and immediate post-delivery services often have well documented perceptions of maternal healthcare acceptability [5–7]. However, maternal healthcare acceptability remains a controversial and complex concept within wider scientific community including maternal health professionals, public health specialists, social psychologists and anthropologists.

The complexity of maternal healthcare acceptability has made it difficult for stakeholders to agree on a precise definition [1, 3, 8]. Nevertheless, most authors agree that the concept of healthcare acceptability is best expressed in overreaching terms such as beliefs, expectations, experiences, attitudes, trust, confidentiality and support [8–10]. Most of these terms have been well described [11, 12] and it is beyond the scope of this study to address each term individually.

Maternal healthcare acceptability is influenced by how women interact with the healthcare providers, the healthcare system and the community [9, 10, 13–15]. Negative maternal healthcare acceptability may ensue when healthcare providers shout or display inappropriate attitudes such as abuse, disrespect, indecency, meanness or mistreatment towards patients [4, 5, 14, 15]. Patients’ perceptions of acceptability may be influenced by facility cleanliness or by policies that directly affect pregnant women including working hours, ambulance service and assistance in birth registration or accessing child grants [6, 16]. Pregnant women also interact with their communities and may experience negative health effects if they are stigmatized or not supported by the father of the child, family and friends [7, 15, 17].

Practically, most stakeholders agree that healthcare acceptability is a key factor in assessing the quality of healthcare services [2, 3, 8]. Some researchers have advocated that healthcare acceptability should be evaluated both retrospectively and prospectively but were largely unclear on the methods of measuring healthcare acceptability [2, 3]. To the best of our knowledge, no tools currently exist to measure maternal healthcare acceptability at institutional, health district, national or international levels. Thus, this study aimed (1) to propose a practical definition of maternal healthcare acceptability; and (2) to develop a tool to retrospectively and prospectively measure the acceptability of maternal healthcare from patients’ perspective at a selected health sub-district in South Africa.

## Methods

We applied the techniques of developing measurement tools, including (1) concept development; (2) specification of concept constructs; (3) selection of indicators; (4) formation of indices; (5) measurement tool/scale construction; and (6) testing of reliability, validity and practicability [18–20].

### Concept development

**Fig. 1:**
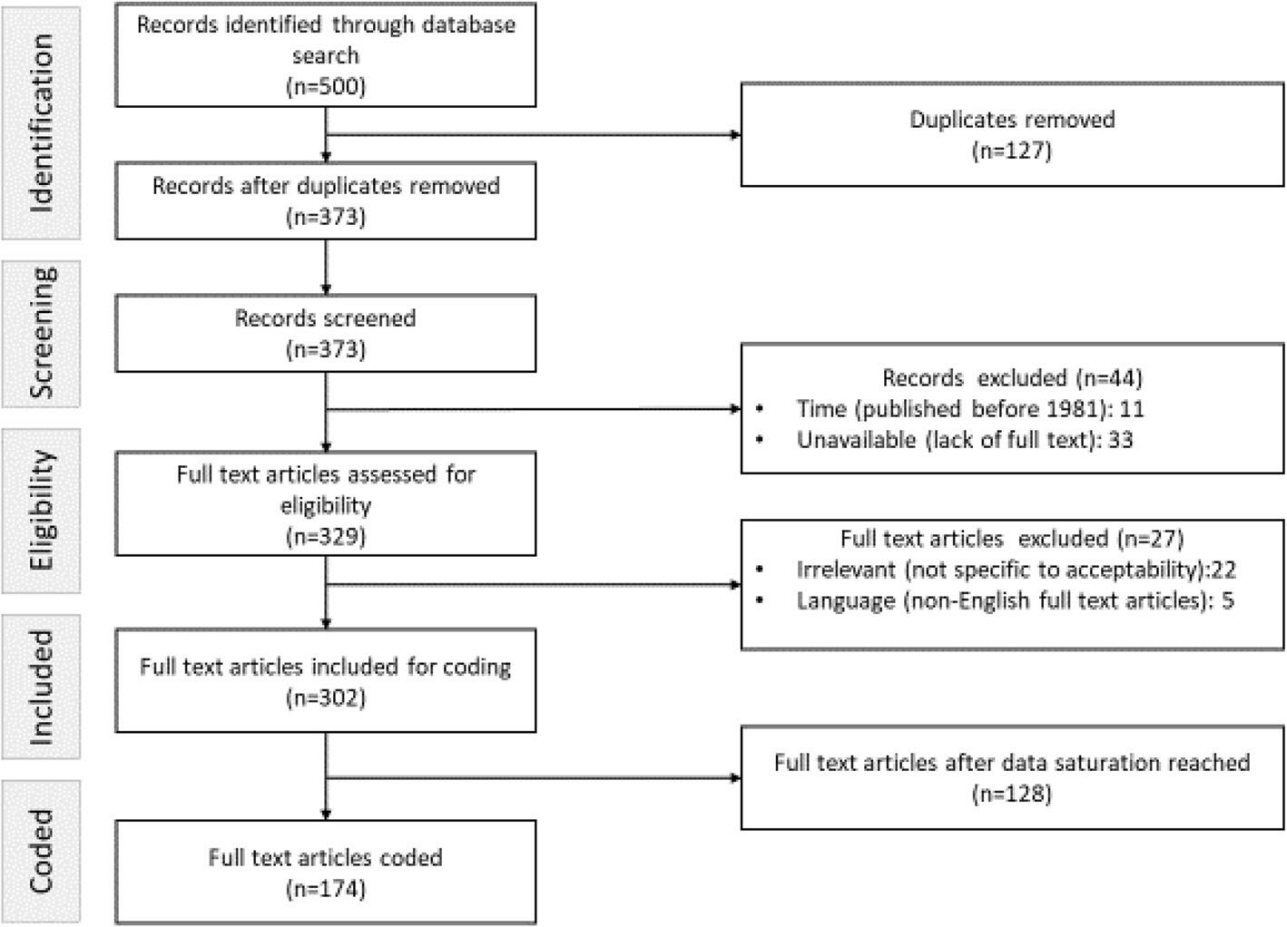
PRISMA-ScR flow diagram

Although the importance of healthcare acceptability is clearly recognized, there is no widely accepted definition of healthcare acceptability [2, 8, 21]. As a starting point, we conducted literature review to identify gaps in defining the concept of healthcare acceptability [22]. We conducted literature search from online databases including MEDLINE/PubMed, Cochrane Library and Google Scholar for relevant articles using “healthcare acceptability”, “concept”, “conceptual framework” and “definition” as key words in different combinations [22]. Different combinations of the key words included for example “healthcare acceptability” AND “definition”, “healthcare acceptability” AND “conceptual framework” or “healthcare acceptability” AND “definition” AND “conceptual framework”. We also applied snowball strategy to check the reference lists of retrieved studies as ‘cited by’ and ‘related’ articles to identify additional sources [22]. We included English literature published between 1981 and 2020. English was the common language of the research team, the concept of healthcare acceptability was first described in 1981 [23] and 2020 was end point of that research project [22]. Out of 500 articles initially retrieved, we retained 174 for thematic content analysis that we imported into Atlas.ti 8.4 software and we coded them until no new information emerged (data saturation) [24]. We followed the Preferred Reporting Items for Systematic reviews and Meta-Analyses extension for Scoping Reviews (PRISMA-ScR) flow diagram (Fig. 1). We then proposed definition and conceptual framework of healthcare acceptability that can be applied to various healthcare contexts including maternal health [22].

Thereafter, we proceeded by conducting a Delhi study in attempt to build a consensus on both proposed definition and conceptual framework of healthcare acceptability concept [25]. We prepared open-ended and rating scale questions for experts to provide their input by modifying the Appraisal of Guidelines for Research & Evaluation II (AGREE II) instrument and a score of 80% was set to indicate the consensus [25]. The questionnaire was piloted and refined before it was sent to the participants [25]. We recruited a sample size of five to ten participants from each of four groups of experts namely: (1) patients; (2) healthcare providers; (3) healthcare researchers; and (4) healthcare managers/policy makers in line with sample size recommendation for Delhi studies [26]. Expert was defined as a person holding a master’s or higher degree or who had knowledge, skills, experience or had published on this topic [25]. Despite our effort to recruit the participants globally, we obtained 34 who completed two rounds of the Delphi study. Out of those 34 experts, 28 came from South Africa, two from the United Kingdom and one from Canada, Lesotho, Rwanda and Zambia respectively [25].

The data collection was semi-anonymous with only the principal investigator (PI) aware of the identity of participants [25]. We conducted the Delphi process in two rounds with the outcomes from the first round informing the second round [25]. The experts reached consensual definition and conceptual framework of healthcare acceptability applicable to varied healthcare disciplines including maternal health [25].

### Specification of concept constructs

**Fig. 2:**
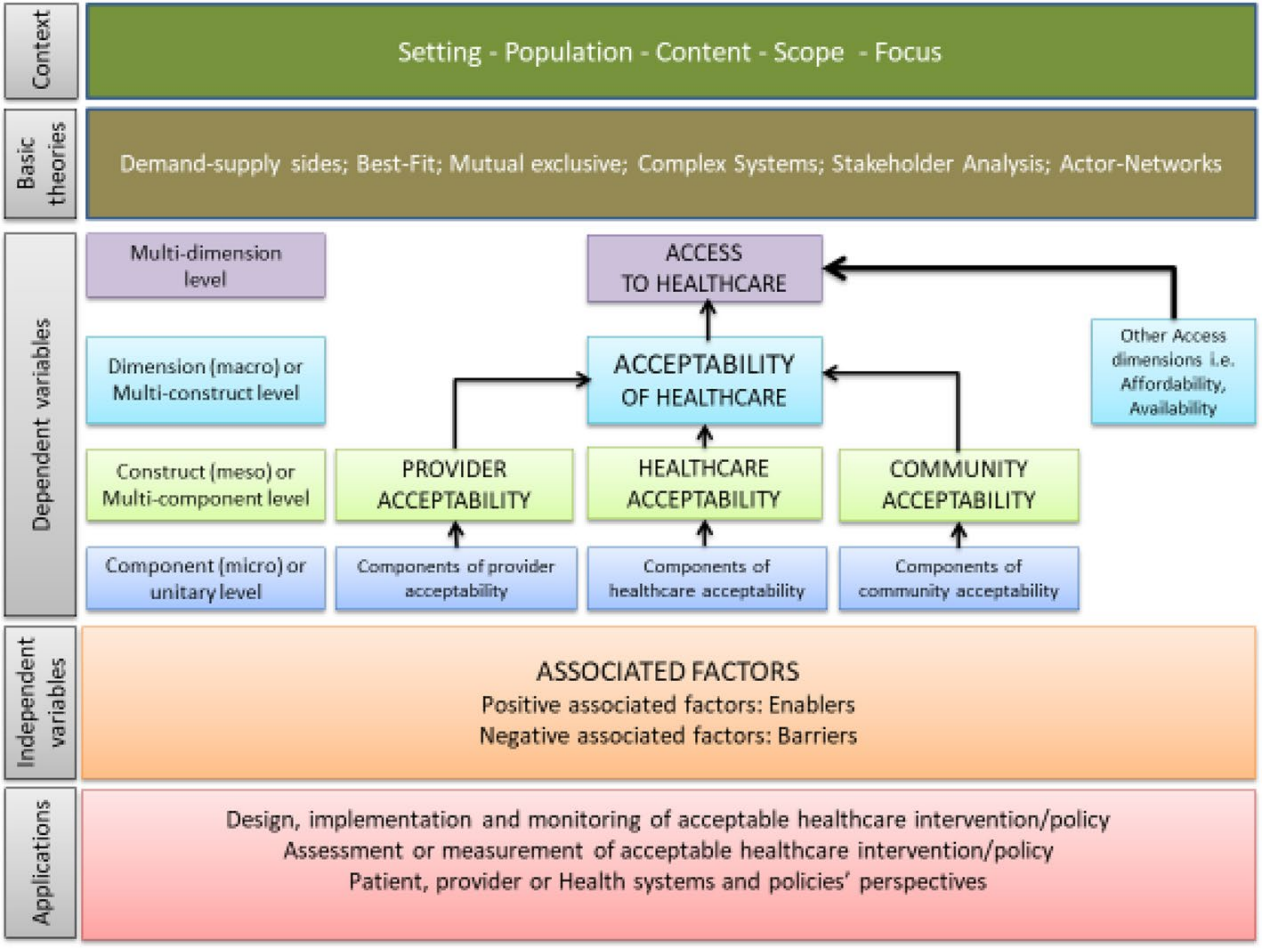
Conceptual framework of healthcare acceptability

Healthcare acceptability is widely considered as one of the dimensions of access to healthcare [9, 27, 28]. Various studies have proposed different constructs of healthcare acceptability^.^ [1–3, 9, 10]. We considered three constructs of acceptability including (1) patient-provider; (2) patient-healthcare system; and (3) patient-community as originally described by Gilson et al. [10]. The “Provider acceptability” or “Provider” construct reflected interactions between patients [mothers] and healthcare providers. The “Healthcare acceptability” or “Healthcare” construct implied interactions between patients [mothers] and the healthcare system or policies. Finally, the “Community acceptability” or “Community” construct indicated interactions between patients [mothers] and the community. Figure 2 shows the conceptual framework of healthcare acceptibility applicable to various healthcare services including maternal healthcare [22, 25]. The proposed framework clearly specifies the constructs of maternal healthcare acceptability.

### Selection of indicators

We selected indicators for each construct that purposely explained the concept of maternal healthcare acceptability in line with scale development theories [18, 19]. The terms “indicator”, “variable”, “component” and “item” are often used interchangeably in the literature on indices development [18, 20, 29, 30]. Similarly, we referred to these terms equivalently. Indicators across constructs were re-scaled to carry the same weight [20]. Indicators were scored using ordinal numbers with similar intervals between scores [20]. Favorable responses scored the highest, while the least favorable response scored lowest and neutral responses scored in the middle [20]. Thus, we rescaled the indicators with scores ranging from 1 to 3, with 1 being the lowest; 2 being intermediate or neutral and 3 being the highest score.

We identified 25 indicators from the questionnaire used previously by a larger Researching Equity and Access in Health Care (REACH) study, which evaluated maternal healthcare acceptability at the same health sub-district in 2008/2009 [1, 31]. The sample size was calculated based on expected use of maternal health services (χ2 Goodness of Fit test, 80% power, medium effect size) [31]. There were three obstetric healthcare facilities selected using the probability proportional to size methodology [1]. At each facility, the researchers interviewed a number of women proportional to the number of deliveries and a total of 359 women participated in that study over 2008/2009 period [1].

In 2020/2021, the principal investigator (PI) used the same REACH questionnaire to collect primary data on maternal healthcare acceptability from the same health sub-district. REACH questionnaire is accessible by clicking on this link: https://osf.io/hcs7d/. The similar number of 359 women aged 18 years old and older seeking maternal health services were interviewed applying the principles of matched sample size at the same facilities where the REACH study was conducted in 2008/2009. Thus, the sample sizes in the two surveys had the same number of participants who were similarly distributed across health facilities.

Very few (1.77%) missing values were recorded in the secondary database and these were handled as neutral scores. There were no missing values in the primary database. The constructs and indicators are described in Table 1.

**Table 1:** Description of constructs and indicators used to measure maternal healthcare acceptability in a sub-district of Johannesburg, South Africa

Construct	Indicator	Description
Provider	P1	The doctors and nurses (health workers) explained what to expect when giving birth
	P2	It is a problem that the health workers DO NOT speak my language
	P3	Was your privacy respected?
	P4	The health workers understood the difficulty of being in labour and assisted me where possible
	P5	Were you offered fluids?
	P6	I DID NOT receive sufficient pain relief during my labour
	P7	In this clinic are you able to talk to the doctors or nurses in private?
	P8	The health workers were too busy to listen to my problems
	P9	Were you shouted at during labour?
	P10	Were you ever hit, slapped or pinched during labour?
	P11	Some staff DO NOT treat patients with sufficient respect
	P12	The health workers I saw cared about me
Healthcare	H1	The facilities (including waiting area and toilets) are dirty
	H2	Were you allowed to have a companion during your labour?
	H3	How satisfied were you with the service today?
	H4	Did you get referred for follow up care for you and the baby?
	H5	For birth registration, did you get all the necessary documents?
	H6	Were you told about the child-care grant & where to go for the childcare grant if you qualify?
	H7	Do you think your delivery was well-managed?
Community	C1	I had all the support that I needed during my pregnancy from the father of the child
	C2	I had all the support that I needed from my family
	C3	I had all the support that I needed from my friends
	C4	I received financial help from the father of the child
	C5	I received financial help from my family
	C6	I received financial help from my friends

### Formation of index

We used two different methods to develop the maternal healthcare measurement tool. Firstly, we conducted factor analysis to create acceptability indices [18, 19] and simple arithmetic equation for practical consideration where factor analysis was not suitable [20]. Factor analysis is an accepted method of reducing correlated variables/indicators into fewer factors explaining the most variability in a correlation matrix [18]. Factor analysis was suitable for developing maternal healthcare acceptability indices on secondary database but not on primary database. As a matter of fact, exploration factor analysis failed to retain three factors respectively representing provider, healthcare and community indices. In this instance, we considered simple arithmetic equation as an alternative method for developing acceptability indices [20]. Secondary and primary databases on acceptability of maternal healthcare were respectively collected in 2008/2009 as part of REACH study and in 2020/2021 as part of the principal investigator’s PhD research project from the same selected health sub-district in South Africa. The use of secondary data analysis was justified by the fact that none of articles on REACH study considered the development of maternal healthcare acceptability measurement tool which was the purpose of this manuscript.

#### Factor analysis

##### Suitability

Factor analysis is widely used to create indices from multi-dimensional data. [18, 19, 29]. However, this method would be applicable based on the suitability characteristics namely: (1) sample size > 250 participants; (2) Bartlett’s test *p*-value < 0.05; and (3) Kaiser–Meyer–Olkin (KMO) > 0.50 [18].

##### Exploratory factor analysis

We computed exploratory factor analysis and retained three factors to predict “Provider”. “Healthcare” and “Community” indices. Retained factors should have an Eigenvalue > 1.0 with explained cumulative variability of 60% or more [18]. Factor rotation, scatter plots of the loadings and score variable were used to improve factor loadings and enhance the visualizations of retained factors [19].

##### Hypothesis formulation

Exploratory factor analysis led to the formulation of a hypothesis that a certain number of indicators would explain the three retained factors to guide the development of a structural equation model (SEM).

##### Confirmation factor analysis

Following the exploratory factor analysis, we performed a confirmatory factor analysis and built a SEM using loadings from the retained factors. Then, the model was confirmed by running a goodness of fit test and regression to test the relationships depicted in the SEM [18].

#### Simple arithmetic equation

##### Suitability

We performed simple arithmetic equation to create maternal healthcare acceptability indices on the primary database which was not suitable for factor analysis. Simple arithmetic equation allowed to create acceptability indices by performing the four basic arithmetic operations including addition, subtraction, multiplication and division. This method can be used if users lack of advanced statistical knowledge or software, or when factor analysis is unsuitable [20].

##### Normalizing indicators

We normalized indicators, so that each of the three constructs had equal numbers of indicators and carried the same weights [20, 29].

##### Simple arithmetic calculation

Indices for each construct were calculated as a mean indicator score in each construct. The scores for each construct were then averaged to obtain an overall index of maternal healthcare.

*Formula for simple arithmetic equation*. Formula used to calculate acceptability indices using simple arithmetic equation are provided below.

$$\begin{array}{cc}Provider\ acceptability\ index=\frac{\text{n[max(}P_k\text{ )]+1-}\sum_{i=1}^nP_i}{n\lbrack\text{max(}P_k\text{ )-min(}P_k\text{ )]+1}}\times100&\mathrm{for}\;\mathrm{any}\;k\in\lbrack1,n\rbrack\end{array}$$

$$\begin{array}{cc}Healthcare\ accept\ index=\frac{\text{n[max(}H_k\text{ )]+1-}\sum_{i=1}^nH_i}{n\lbrack\text{max(}H_k\text{ )-min(}H_k\text{ )]+1}}\times100&\mathrm{for}\;\mathrm{any}\;k\in\lbrack1,n\rbrack\end{array}$$

$$\begin{array}{cc}Community\ accept\ index=\frac{\mathrm n\lbrack\max(C_k)\rbrack+1-\sum_{i=1}^nC_i}{n\lbrack\max(C_k)-\min(C_k)\rbrack+1}\times100&\mathrm{for}\;\mathrm{any}\;k\in\lbrack1,n\rbrack\end{array}$$

$$Maternal\ healthcare\ acceptability\ index\ =\ \frac{\text{(}Provider\ acceptability\ index\ +\ healthcare\ acceptability\ index\ +\ community\ acceptability\ index\text{)}}3$$

### Maternal healthcare measurement tool

We proposed two different measurement tools to assess the maternal healthcare acceptability, one for each of the recommended methods: factor analysis and simple arithmetic equation at health institutional level.

#### Maternal healthcare acceptability measurement tool using factor analysis

Table S1 shows the maternal healthcare acceptability measurement tool using factor analysis.

#### Maternal healthcare acceptability measurement tool using simple arithmetic equation

Table S2 shows the maternal healthcare acceptability measurement tool using simple arithmetic equation.

### Reliability, validity and practicability

Reliability. validity and practicability are key considerations when developing a measurement tool [18].

#### Factor analysis

We used Cronbach’s alpha to assess reliability and alpha values > 0.70 were considered ideal while values between 0.45 to 0.70 were deemed acceptable [32]. We also conducted a confirmatory factor analysis to test the fitness and validity of the SEM [33]. Convergent validity was assessed by the average variance extracted (AVE) calculated from the CFA output [34]. Each construct was evaluated against its correlation with other constructs and each factor AVE greater than 0.5 to indicates good convergent validity [34]. Discriminant validity was evaluated by the maximum shared variance (MSV) lower than AVE [34]. AVE was calculated as the sum of the square of factor loadings divided by the number of items, whereas the MSV was calculated as the square root of the AVE for each construct [34]. Factor analysis was regarded practicable in settings with availability of appropriate statistical analysis software and knowledge.

#### Simple arithmetic equation

To ensure reliability and validity, we used an equal number of indicators with the same weight, scores and ranks within each construct, negating the need for further normalization or robustness techniques [20, 29]. We considered simple arithmetic equation to be an alternative practicable approach in settings where advanced statistical analysis knowledge or software were unavailable.

## Results and validation

### Practical definition of acceptability of maternal healthcare

We invited 92 experts to provide their inputs on a proposed definition of maternal healthcare acceptability. Of the invited 92, 47 experts submitted answers in the first round of questions (51.1% response rate) and 34 participated in both Delphi rounds (27.6% loss to follow up). These experts refined initial proposed definition and agreed that maternal healthcare acceptability could be defined as *“a multi-construct concept describing the nonlinear cumulative combination in parts or in whole of experienced or anticipated maternal healthcare from the relevant patients/participants, communities, providers/researchers or healthcare systems' managers and policy makers' perspectives in a given context".* Of the 34 experts who participated in two Delphi surveys, 29.4% were expert-patients, 26.5% healthcare researchers, 23.5% healthcare providers and 20.6% were healthcare managers/policy makers. Most of the experts (82.4%) resided in South Africa at the time of the study and 50% were women. We selected 11 experts who validated the final definition of maternal healthcare acceptability.

### Practical measurement tool to assess acceptability of maternal healthcare: findings

We used two methods to develop the practical measurement tool to assess the acceptability of maternal healthcare: factor analysis and simple arithmetic equation.

#### Factor analysis

##### General information

We used secondary data collected for 359 women attending maternal healthcare services in 2008/2009 as part of REACH study [1]. In total, we counted 25 indicators with 12, 7 and 6 indicators representing provider (P), healthcare (H) and community (C) constructs respectively (Table 1).

##### Suitability

The KMO value of 0.645 and the *p*-value < 0.01 together with a sample size of > 250 participants indicated suitability for factor analysis (Table S1).

##### Exploratory factor analysis

**Fig. 3:**
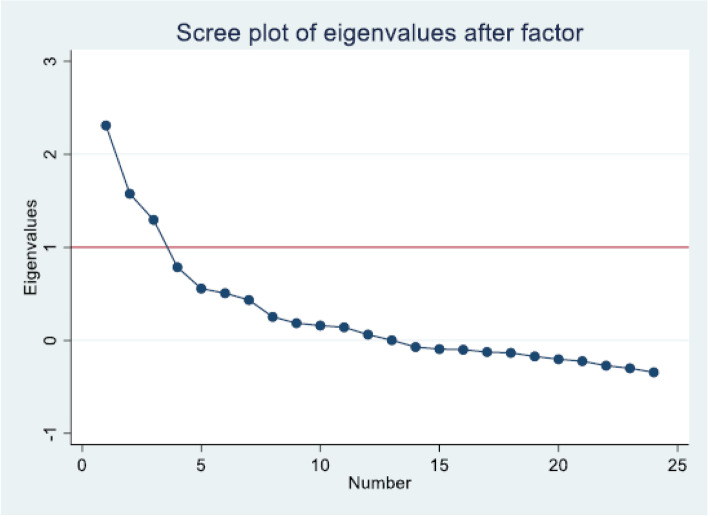
Scree plot of eigenvalues after factor

We initially included all 25 indicators in exploratory factor analysis and noted that the second factor was cross-loading on P and H indicators (P11, P12 and H3). We removed H3 and re-ran exploratory factor analysis on the remaining 24 indicators (Table 2). We obtained 3 factors without cross-loading and with eigenvalues ≥ 1. These factors were retained and cumulatively explained 83.1% of the correlation matrix variability. Factor loadings (un-rotated as well as orthogonal and oblique rotated) yielded similar results. The scree plot of eigenvalues confirmed the retention of 3 factors (Fig. 3).

**Table 2:** Exploratory factor analysis output (24 indicators) used to identify important indicators for developing a tool to measure acceptability of maternal healthcare

**Factor analysis/correlation**		**Number of obs**	** = **	**359**
Method: principal factors		Retained factors	=	3
Rotation: (unrotated)		Number of params	=	69
Factor	**Eigenvalue**	**Difference**	**Proportion**	**Cumulative**
Factor1	2.31	0.73	0.37	0.37
Factor2	1.58	0.28	0.25	0.62
Factor3	1.30	0.51	0.21	0.83
Factor4	0.79	0.23	0.13	0.96
Factor5	0.56	0.05	0.09	1.05
Factor6	0.51	0.07	0.08	1.13
Factor7	0.43	0.18	0.07	1.20
Factor8	0.25	0.07	0.04	1.24
Factor9	0.19	0.03	0.03	1.27
Factor10	0.16	0.02	0.03	1.29
Factor11	0.14	0.08	0.02	1.32
Factor12	0.06	0.06	0.01	1.33
Factor13	0.00	0.07	0.00	1.33
Factor14	-0.07	0.02	-0.01	1.31
Factor15	-0.09	0.01	-0.01	1.30
Factor16	-0.10	0.02	-0.02	1.28
Factor17	-0.12	0.01	-0.02	1.26
Factor18	-0.13	0.04	-0.02	1.24
Factor19	-0.17	0.03	-0.03	1.21
Factor20	-0.20	0.02	-0.03	1.18
Factor21	-0.22	0.05	-0.04	1.15
Factor22	-0.27	0.03	-0.04	1.10
Factor23	-0.30	0.04	-0.05	1.05
Factor24	-0.34		-0.05	1.00

To facilitate the naming of factors, we considered the factor loadings ≥ 0.4. Factor 1 was heavily loaded on H4, H5 and H6, and was named “Healthcare system and policy” or “Healthcare”. Factor 2 was strongly loaded on P9, P11 and P12 and was named “Healthcare provider” or “Provider”. Factor 3 was sharply loaded on C1, C4 and C5 and was named “Community support” or “Community (Table 3). These nine factors: P9, P11, P12, H4, H4, H6, C1, C4 AD C5 were statistically correlated (KMO = 0.615 and *p* value < 0.001).

**Table 3:** Factor loadings (≥ 0.4)

Variable	Factor1	Factor2	Factor3	Uniqueness
P1				0.91
P2				0.90
P3				0.90
P4				0.83
P5				0.85
P6				0.87
P7				0.86
P8				0.93
P9		-0.47		0.76
P10				0.93
P11		-0.44		0.75
P12		0.67		0.51
H1				0.97
H2				0.98
H4	0.82			0.30
H5	0.85			0.27
H6	0.53			0.70
H7				0.93
C1			0.60	0.58
C2				0.97
C3				0.97
C4			0.67	0.47
C5			-0.46	0.73
C6				0.99

##### Confirmation factor analysis: graphical representation of structural equation model

**Fig. 4:**
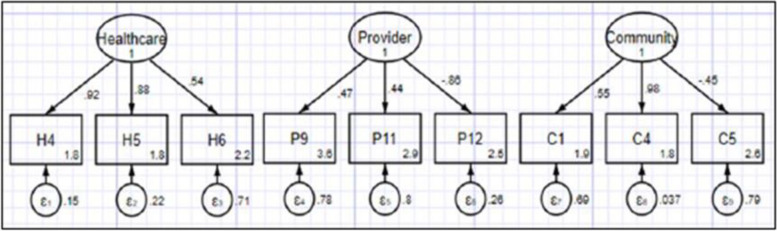
Structural equation model

We built the SEM by applying the 3 retained factors with loadings ≥ 0.4. Figure 4 shows the SEM of maternal healthcare acceptability constructs and their corresponding indicators.

##### Confirmation factor analysis: maternal healthcare acceptability indices in 2008/2009

We used individual proportion and cumulative variability of the 3 retained factors to determine indices for maternal healthcare acceptability. We noted quite poor levels of acceptability particularly with provider and healthcare indices below 50%. The community index was 68% with overall maternal healthcare index of 52. 65% (Table 4).

**Table 4:** Maternal healthcare acceptability indices in 2008/2009 for a selected health sub-district in South Africa

Variable	Obs	Mean	Std. Dev	Min	Max
Provider index (1–100%)	359	32.93	14.31	17	100
Healthcare Index (1–100%)	359	48.33	24.31	25	100
Community Index (1–100%)	359	68.25	14.32	17	100
Maternal healthcare index (1–100%)	359	52.65	11.21	50	100

##### Confirmation factor analysis: structural equation model fitness

We assessed fitness of the SEM using the Chi square P value, the Root Mean Square Error of Approximation (RMSEA), the comparative fit index (CFI), the Tucker-Lewis index (TLI), the Standardized root mean residual (SRMR) and the Coefficient of determination (CD). The results showed a good fit model (Table 5).

**Table 5:** Fitness of structural equation model

Fit statistic	Value	Description
**Likelihood ratio**		
chi2_ms (27)	51.47	model vs. saturated
*p* > chi2	0.003	
chi2_bs (36)	874.102	baseline vs. saturated
*p* > chi2	0.000	
**Population error**		
RMSEA	0.05	Root mean squared error of approximation
90% CI, lower bound	0.029	
upper bound	0.071	
*p* close	0.461	Probability RMSEA < = 0.05
**Baseline comparison**		
CFI	0.971	Comparative fit index
TLI	0.961	Tucker-Lewis index
**Size of residuals**		
SRMR	0.055	Standardized root mean squared residual
CD	0.999	Coefficient of determination

##### Confirmation factor analysis: testing reliability and validity

Cronbach’s alpha for healthcare factor indicated strong reliability (> 0.70) and acceptable reliability for factors 2 and 3 (> 0.45). Composite reliability (CR) indicated strong construct reliability for healthcare factor (> 0.70) and weak reliability for the provider and community factors (< 0.70). Construct validity was confirmed by high factor loadings factors (ranging from 0.4 to 0.9). We assessed convergent and discriminant validity using Average Variance Extracted (AVE) and Maximum Shared Variance (MSV). The healthcare factor had a very good convergent validity with AVE value > 0.50. The provider and community factors had borderline convergent validity with AVE values of 0.38 and 0.49 respectively. The model achieved the discriminant validity because the AVE value for each construct was higher than the MSV value for the same construct (Table 6).

**Table 6:** Reliability and validity of each factor, with respective indicators, used to create indices to measure acceptability of maternal healthcare

Factors	Indicators	Factor loading (standardized)	Cronbach’s α	CR	AVE	MSV
Healthcare	H4	0.92	0.81	0.84	0.64	0.0004
	H5	0.88				
	H6	0.54				
Provider	P9	0.47	0.54	0.001	0.38	0.0004
	P11	0.44				
	P12	-0.86				
Community	C1	0.56	0.67	0.44	0.49	0.0001
	C4	0.98				
	C5	-0.45				

##### Confirmation factor analysis: hypothesis testing of structural equation model

The SEM hypothesis testing confirmed that all items and corresponding factors were associated. The null hypothesis (H_o_) was rejected in all instances with the *p*-value < 0.01 and none of the 95% confidence intervals included zero. Table 7 shows the results of the hypothesis testing with standardized regression coefficients, t-values and *p*-values.

**Table 7:** SEM hypothesis testing results

Relationships	Stand. Regr. Coef	t-values	*p*- values	[95% CI]	H_o_
H4 → Healthcare	0.39	14.73	0.001	0.34–0.44	Rejected
H5 → Healthcare	0.52	20.46	0.001	0.47–0.57	Rejected
H6 → Healthcare	0.12	6.86	0.001	0.08–0.15	Rejected
P9 → Provider	-0.299	-9.40	0.001	-0.36—-0.24	Rejected
P11 → Provider	-0.231	-8.54	0.001	-0.28—-0.18	Rejected
P12 → Provider	1.10	20.03	0.001	0.99–1.20	Rejected
C1 → Community	0.47	14.74	0.001	0.40–0.53	Rejected
C4 → Community	0.50	17.48	0.001	0.44–0.55	Rejected
C5 → Community	-0.21	-9.75	0.001	-0.26—-0.17	Rejected

#### Simple arithmetic equation

##### General information

We used primary data collected on 359 women in 2020/2021 on maternal health services acceptability from a selected health sub-district in South Africa. We considered three latent variables or constructs (provider, healthcare and community) to represent maternal healthcare acceptability. Although we identified 25 indicators for each construct (Table 1), we only included the first six indicators per construct so that all constructs had the same number of indicators.

##### Suitability

We ensured that all indicators were normalized by re-scaling them into the same standard scale. Each indicator had three possible responses, ranging from 1 to 3.

##### Determining maternal healthcare acceptability indices

We applied simple arithmetic equation to create additive composite indices of maternal healthcare acceptability (Table 8).

**Table 8:** Maternal healthcare acceptability indices in 2020/2021 for a selected health sub-district in South Africa

Variable	Obs	Mean	Std. Dev	Min	Max
Provider index (1–100)	359	63.25	16.53	8	100
Healthcare index (1–100)	350	63.46	15.96	8	100
Community index (1–100)	358	89.09	20.01	8	100
Maternal healthcare index (1–100)	349	71.86	10.94	25	97

##### Reliability and validity

We assumed that the reliability and validity of using simple arithmetic equation to measure maternal healthcare acceptability would be achieved by normalization of indicators. We identified these indicators based on our deep understanding of the definition and conceptual framework of maternal healthcare acceptability.

### Practical measurement tool to assess acceptability of maternal healthcare: application

To simplify practical, policy making and research applications by a wider ranges of health practitioners in the field of maternal health, the proposed acceptability measurement tool was completed using factor analysis and simple arithmetic equation as an illustration (Table 9 and 10). Both methods satisfied the minimum general conditions and suitability criteria pre-established during the development phase of the measurement tool for maternal healthcare acceptability. Ideal and acceptable values for SEM fitness, reliability and validity were indicated in line with existing literature (Table 9). A list of indicators for each construct was presented with a statement for data attachment as appendix not only for transparency but for further analysis by different researchers with interest in this field (Table 9 and 10).

**Table 9:** Healthcare acceptability measurement tool using factor analysis

Healthcare acceptability measurement tool using factor analysis							
Health Institution: Sub-District of Johannesburg							
Service: Maternal healthcare							
Data collection period:2008/2009							
**General information**							
		Observed			Reference		
Number of included indicators for “Provider” construct		12			Minimum 3		
Number of included indicators for “Healthcare” construct		6			Minimum 3		
Number of included indicators for “Community” construct		6			Minimum 3		
Number of indicator response options (scale)		3			Minimum 3		
Number of participants (sample size):		359			≥ 250		
**Suitability**							
Correlation matrix Bartlett’s test *p*-value		˂ 0.01			< 0.05		
Kaiser–Meyer–Olkin (KMO) measure of sampling adequacy		0.64			> 0.50		
**Exploratory factor analysis**							
Number of retained factors^a^		3			3		
Percentage of variability explained		0.83.1			≥ 0.60		
**Confirmation factor analysis**							
**Structural Equation Model (SEM) fitness**							
chi-square *p*-value		0.003			< 0.05		
Root mean square error of approximation (RMSA)		0.05			< 0.5 (ideal); (0.5–0.8): acceptable		
Comparative fit index (CFI)		0.97			> 0.95 (ideal); (> 0.90): acceptable		
Tucker-Lewis index (TLI)		0.96			> 0.95 (ideal); (> 0.90): acceptable		
Standardized root mean residual (SRMR)		0.055			< 0.05 (ideal); (0.05–0.10): acceptable		
**Reliability**							
Composite reliability (CR)					> 0.70 (ideal); (0.45 – 0.70) (acceptable)		
Provider		0.001					
Healthcare		0.81					
Community		0.44					
Cronbach’s alpha value (Reliability)					> 0.70 (ideal); (0.45—70): acceptable		
Provider		0.54					
Healthcare		0.81					
Community		0.67					
**Validity**							
Convergent validity (AVE)					> 0.50		
Provider		0.38					
Healthcare		0.64					
Community		0.49					
Discriminating validity (		AVE	MSV		AVE > MSV		
Provider		0.38	0.0004				
Healthcare		0.64	0.0004				
Community		0.49	0.0001				
**Acceptability index**							
Scale range (1–100%)		Mean		Std.dev	Min	Max	
Provider index		32.93		14.31	17	100	
Healthcare Index		48.33		24.31	25	100	
Community Index		68.25		14.32	50	100	
Maternal healthcare index (1–100%)		52.65		11.21	50	100	
**List of indicators included**							
**Provider construct variables**	**Healthcare construct variables**	**Community construct variables**					
The doctors and nurses (health workers) explained what to expect when giving birth	The facilities (including waiting area and toilets) are dirty	I had all the support that I needed during my pregnancy from the father of the child					
It is a problem that the health workers DO NOT speak my language	Were you allowed to have a companion during your labour?	I had all the support that I needed from my family					
Was your privacy respected?	Did you get referred for follow up care for you and the baby?	I had all the support that I needed from my friends					
The health workers understood the difficulty of being in labour and assisted me where possible	For birth registration, did you get all the necessary documents?	I received financial help from the father of the child					
Were you offered fluids?	Were you told about the child-care grant & where to go for the childcare grant if you qualify?	I received financial help from my family					
I DID NOT receive sufficient pain relief during my labour	Did you get referred for follow up care for you and the baby?	I received financial help from my friends					
In this clinic are you able to talk to the doctors or nurses in private?							
The health workers were too busy to listen to my problems							
Were you shouted at during labour?							
Were you ever hit, slapped or pinched during labour?							
Some staff DO NOT treat patients with sufficient respect							
The health workers I saw cared about me							
**Confirmation of dataset availability**						Yes	√

^a^If the number of retained factors during exploratory factor analysis, is different than 3 representing provider, healthcare and community respectively, then consider to use arithmetic analysis method to calculate maternal healthcare acceptability

**Table 10:** Healthcare acceptability measurement tool using simple arithmetic equation

Healthcare acceptability measurement tool using simple arithmetic analysis						
Health Institution: Sub-District of Johannesburg						
Service: Maternal healthcare						
Data collection period: 2020/2021						
**General information**						
		Observed		Reference		
Number of included indicators for “Provider” construct		6		Minimum 3		
Number of included indicators for “Healthcare” construct		6		Minimum 3		
Number of included indicators for “Community” construct		6		Minimum 3		
Number of indicator response options (scale)		3		Minimum 3		
Number of participants (sample size)		359		≥ 3 (nber of items x nber of scale)		
**Suitability**						
Normalized indicators		Yes		Yes		
Equal number of indicators per construct		Yes		Yes		
**Acceptability index**						
Scale range (1–100%)		Mean	Std.dev	Min	Max	
Provider index		63.25	16.53	8	100	
Healthcare Index		63.46	15.96	8	100	
Community Index		89.09	20.01	8	100	
Maternal healthcare index		71.86	10.94	25	97	
**List of indicators included**						
**Provider construct variables**	**Healthcare construct variables**	**Community construct variables**				
The doctors and nurses (health workers) explained what to expect when giving birth	The facilities (including waiting area and toilets) are dirty	I had all the support that I needed during my pregnancy from the father of the child				
It is a problem that the health workers DO NOT speak my language	Were you allowed to have a companion during your labour?	I had all the support that I needed from my family				
Was your privacy respected?	How satisfied were you with the service today?	I had all the support that I needed from my friends				
The health workers understood the difficulty of being in labour and assisted me where possible	Did you get referred for follow up care for you and the baby?	I received financial help from the father of the child				
Were you offered fluids?	For birth registration, did you get all the necessary documents?	I received financial help from my family				
I DID NOT receive sufficient pain relief during my labour	Were you told about the child-care grant & where to go for the childcare grant if you qualify?	I received financial help from my friends				
**Confirmation of dataset availability**				Yes		√

#### Maternal healthcare acceptability measurement tool using factor analysis

Table 9 shows completed maternal healthcare acceptability measurement tool using factor analysis.

#### Maternal healthcare acceptability measurement tool using simple arithmetic equation

Table 10 shows completed maternal healthcare acceptability measurement tool using simple arithmetic equation.

## Discussion

Defining and measuring acceptability of healthcare remains a challenge through existing public health literature [2, 3, 8, 35]. Nevertheless, our study upholds experts’ consensual definition of maternal healthcare acceptability and the results revealed practical measurement tools to assess retrospectively and prospectively acceptability of maternal healthcare.

We concurred with existing literature that acceptability [cultural access] remains neglected and poorly defined compare to other healthcare access dimensions such as affordability [financial access] and availability [geographical access] [3, 35]. In our study, we used expert knowledge to reach a consensual definition of maternal healthcare acceptability, namely *“a multi-construct concept describing the nonlinear cumulative combination in parts or in whole of experienced or anticipated maternal healthcare from the relevant patients/participants, communities, providers/researchers or healthcare systems' managers and policy makers' perspectives in a given context.” ".* This definition was validated and recommended by selected experts in line with guidance on conducting and reporting Delphi studies (CREDES) best practices [36].

Furthermore, we agreed with scholars who advocated for the need of a measurement tool to assess retrospectively and prospectively the concept of acceptability of healthcare [2, 3]. In this study, we analyzed both secondary and primary databases to demonstrate retrospective and prospective measurement of maternal healthcare acceptability. In line with known techniques to develop measurement tools [18–20], we explained and demonstrated the processes of constructs specification and indicators selection relating to maternal healthcare acceptability indices.

We applied factor analysis as a preferable method to reduce many indicators into fewer numbers of constructs [18–20, 29] to create maternal healthcare acceptability indices. Through exploratory factor analysis, we retained three factors to predict the acceptability indices. We conducted confirmation factor analysis and developed a structural equation model showing relationships between acceptability constructs their corresponding variables. The fitness tests showed a good fit model achieving good reliability and validity. The regression analysis confirmed the hypothesis with significant relationships between the retained factors and their corresponding variables (*p*-value < 0.01 throughout). These results were consistent with findings from other studies on developing indices using factor analysis method [18, 19].

Unlike most studies on index development applying factor analysis [18, 19, 30, 37], this study suggests simple arithmetic equation as alternative method in case the factor analysis is not suitable. The simple arithmetic equation would also be recommended when appropriate statistical knowledge is missing such as in rural health facilities without biostatisticians. While application of simple arithmetic equation is relatively easy, the reliability and validity of its results are largely based on clear understanding of maternal healthcare acceptability concept and appropriate normalization of the variables [20].

## Limitations

We were limited by a lack of research funding to collect data at national level. This challenge was exacerbated by the Covid-19 pandemic and associated prevention measures and policies limiting our access to women attending maternal healthcare services in a health sub-district of South Africa. Our results can unfortunately not be generalized at provincial, national and international levels. Factor analysis was not suitable for data collected in 2020/2021 and we applied simple arithmetic equation as alternative method. Investigating statistical difference and its magnitude between maternal healthcare acceptability indices generated using factor analysis and those generated using simple arithmetic equations was beyond the scope of this manuscript. Moreover, experts who participated in Delphi surveys resided in a relatively narrow range of countries despite our efforts to recruit global experts. Accordingly, it is difficult to say with certainty that the proposed definition would have universal pertinence.

## Conclusion

We sought to define and develop a practical tool to assess acceptability of maternal healthcare from patients’ perspectives from a selected health sub-district in South Africa. We applied the techniques of developing measurement tool, and we presented a consensual definition and measurement tool to assess maternal healthcare acceptability using factor analysis. We suggest that simple arithmetic analysis may be a suitable alternative if factor analysis is not applicable or if there is a lack of advanced knowledge in statistics. It is important that variables are normalized when using simple arithmetic so that indicators carry the same weight, and each construct is equally represented.

In order to retrospectively and prospectively assess maternal healthcare acceptability, it is advisable to regularly collect information on maternal healthcare acceptability that can be used as secondary or baseline database that will inform the collection of primary data. Furthermore, it requires the same number of indicators that are similarly scaled or normalized with the same method of index formation either factor analysis or simple arithmetic equation to compare or to measure acceptability of healthcare interventions over time at the institutional levels.

Our results complement existing evidence on the concept of healthcare acceptability. We also believe that this study will allow health professionals apply and assess this concept with greater confidence. We expect that researchers from public health, psychology, maternal healthcare, anthropology and other health disciplines will undertake further research at national and international levels to build on these results and shed more light on the concept of maternal healthcare acceptability.

## Supplementary Information

**Additional file 1:** Anonymous questionnaire.

**Additional file 2: Table S1.** Healthcare acceptability measurement tool using factor analysis.

**Additional file 3: Table S2.** Healthcare acceptability measurement tool using simple arithmetic equation.

## Data Availability

This is an Open Access article distributed in accordance with the Creative Commons Attribution Non-Commercial (CC BY-NC 4.0) license, which permits others to distribute, remix, adapt, build upon this work non-commercially, and license their derivative works on different terms, provided the original work is properly cited and the use is non-commercial. To ensure transparency, the data collected and analysed during the current study are publicly available from Open Science Framework (OSF) and can be accessed by using this link: https://osf.io/hcs7d/.
