# Supplementary material for: A tool to define and measure maternal healthcare acceptability at a selected health sub-district in South Africa
Source: BMC Pregnancy Childbirth. 2023 Apr 29;23:302. doi: 10.1186/s12884-023-05475-y (PMC10148523; doi:10.1186/s12884-023-05475-y)
Supplement: Supplementary file 1 — Additional file 1: Anonymous questionnaire. [file 12884_2023_5475_MOESM1_ESM.pdf]

Anonymous questionnaire

STUDY IDENTIFICATION NUMBER	545/2019
PARTICIPANT IDENTIFICATION NUMBER	

You will have to fill the following questionnaire if you decide to join the study. Please do not complete the section if you are unsure of the required information. If you have anu question, I am available to explain all sections.

Essential information (required)

Answering the questions is completely voluntary and participants may leave out any questions that make them feel uncomfortable. This data will be used anonymously in our network and your privacy will be protected to the best of our ability.

MOTHER' S EXIT INTERVIEW reach<br>PhD study ADAPTED QUESTIONNAIRE	
0.1<br>Date of interview	dd / mm / yyyy
0.2<br>Interviewer name	

0.3<br><br>Patient number	
0.4<br><br>Start time of interview	____________:_______________<br>hour min
0.5<br><br>Site (name of facility)	_______________________________________

Instructions for interviewers:

Questions or parts of questions that do not always need to be read out and instructions are in highlighted text.

Skips indicating which questions can be left out are indicated by arrow: ← **Go to**

Unless specifically asked to do so, options do not need to be read out.

SECTION 1: SOCIO - DEMOGRAPHIC AND PARITY BACKGROUND QUESTIONS

READ OUT:

I am going to start by asking you a few questions about you and your household

Now, let start first by asking you a few questions about yourself.

**1.1**<br><br>: How would you describe	African/Black	1
	Coloured	2
	Asian/Indian	3

yourself racially?	White	4
	Other (specify)	
**1.2**<br><br>What was your age at your last birthday?<br><br>Fill in one block only	____________________<br>Year born	____________________<br>Years
**1.3**<br><br>What is your current marital status?<br><br>Tick one block only	Married	1
	Living with partner	2
	Widow/widower	3
	Divorced or separated	4
	Never married (single)	5
	Other (specify)	
**1.4**<br><br>What is YOUR highest level of education?<br><br>Tick one block only	**Type of education**	
	No schooling	1
	Highest grade passed in school (1-12)	2
	Completed diploma/certificate	3
	Completed degree	4
	Other (specify)	**5**
**1.5**<br><br>When you became pregnant, were you employed working or earning money?	**Type of employment**	
	Yes, full-time	1
	Yes, part-time	2
	No	3 ← **Go to 1.9**
**1.6**	**Nature of employment**	
	Self-employed	1

If respondent employed ask:<br><br>Were you self-employed or do you work for someone else?	Employee		2
**1.7**<br><br>Are you on a maternity leave benefit?	Yes		1
	No		0
**1.8**<br><br>Will you be able to return to your same job after your maternity leave?	Yes		1
	No		0
	Don't know		99
**1.9**<br><br>If respondent was not employed ask:<br><br>What are the reasons that you are not employed?<br><br>Tick all that apply "Yes" and others "No"	**Reason**	**Yes**	**No**
	Looking for work	1	0
	Retired or pensioner	1	0
	Sick or injured	1	0
	Pregnant or caring for own children	1	0
	Caring for other children	1	0
	Caring for sick/injured	1	0
	Retrenched	1	0
	Nothing	1	0
	Don't know	99	
	Other (specify)		

Go to 1.10

Let's continue by talking about your household. When I talk about your household, I am including all the people who live in your house and who share the same food with you.

**1.10**<br><br>Who is the head of your household? By this, I mean, who is the person who usually makes the important decisions in the household. Indicate relationship e.g. father, mother not name.	________________________<br>Relationship	**If respon-dent HHH, go to 1.15**
**1.11**<br><br>Code sex of HHH. If not clear ask: What is the sex of your HHH?	Male	1
	Female	2
**1.12**<br><br>Code position in HH of respondent. If unclear, ask:<br><br>What is your position in the household, in relation to the household head such as …read out a few relevant options.	Head/acting head	1
	Husband/wife/partner	2
	Son/daughter/stepchild/adopted child	3
	Brother/sister/step brother/step sister	4
	Father/mother/step father/step mother	5
	Grandparent/great grandparent	6
	Grandchild/great grandchild	7
	Other relative (e.g. in laws or aunt/uncle)	8
	Non-related persons (tenant, boarder, lodger)	9
	Don’t know	99
Tick one block only	Other (specify)	
**1.13**<br><br>What was the age of your HHH i.e. husband / father / mother etc. at his/her last birthday?	________________<br>Year born	________________<br>Years

fill in one block only		

**1.14**<br><br>Does your HHH i.e. husband / father / mother etc. stay with you for at least 2 weeks each month?	Yes	1
	No	0

**1.15**<br><br>What is the highest level of education of your HHH i.e. husband / father / mother etc.?<br><br>Tick one block only	**Type of education of your HHH**	
	No schooling	1
	Highest grade passed in school (1-12)	2
	Completed diploma/certificate	3
	Completed degree	4
	Other (specify)	**5**
**1.16**<br><br>When you became pregnant, was your HHH i.e. husband / father / mother etc. employed?	**Type of employment**	
	Yes, ful-time	1
	Yes, part-time	2
	No	3
	Don’t know	99
**1.17**<br><br>If HHH employed, ask<br><br>Is your HHH i.e. husband / father / mother etc. self-employed or does HE/SHE work for someone else?	**Nature of employment**	
	Self-employed	1
	Employee	2
	Don’t know	99
**1.18**<br><br>Are you on a maternity leave benefit?	Yes	1
	No	0
**1.19**	Yes	1

Go to 1.18 (for No / Don’t know in 1.16)

Will you be able to return to your same job after your maternity leave?	No	0
	Don’t know	99

**1.20**<br><br>If respondent was not employed ask:<br><br>What are the reasons that you are not employed?<br><br>Tick all that apply “Yes” and others “No”	**Reason**	**Yes**	**No**
	Looking for work	1	0
	Retired or pensioner	1	0
	Sick or injured	1	0
	Pregnant or caring for own children	1	0
	Caring for other children	1	0
	Caring for sick/injured	1	0
	Retrenched	1	0
	Nothing	1	0
	Don’t know		99
	Other (specify)		

**1.21**<br><br>Including yourself, how many adults (18 years or older) live in your household? When I talk about your household, I am including all the people who live in your house and who share the same food with you.	
**1.22**<br><br>How many children younger than 18 years live in your household?	

**1.23**<br><br>Does anyone in your household receive a government grant OR income from the government such	**Type of grant**	**Yes**	**No**	**If yes, number received**
	Unemployment insurance (UIF)	1	0	
	Worker’s compensation	1	0	
	State old age pension	1	0	
	Disability grant	1	0	

as…….read out each option and tick yes or no.<br><br>IF YES ask:<br><br>How many of each type of grant is received (i.e. how many people receive each?)	Child support grant	1	0	
	Care dependency grant	1	0	
	Foster care grant	1	0	
	Grant in aid	1	0	
	Social relief	1	0	
	Other	1	0	
	Don't know		99	

**1.24**<br><br>Where were you born? READ OUT I know this is a sensitive question to ask at this stage, but we are asking because we want to see if health services treat South Africans differently to those who are not from South Africa.	South Africa	1
	Other (specify)	2

If other go to 1.26

**1.25**<br><br>If respondent born in South Africa, ask<br><br>Which province were you born in?<br><br>Use current province borders	Western Cape	1
	Eastern Cape	2
	Northern Cape	3
	Free State	4
	KwaZulu-Natal	5
	North West	6
	South Africa	7
	Mpumalanga	8

	Limpopo	9
	Don't Know	99

**1.26**<br><br>If respondent not born in South Africa, ask:<br><br>Do you have a South African ID document?	Yes	1
	No	0
**1.27**<br><br>Are you covered by a Medical Aid or any scheme that helps you pay for health-care services or medicines?	Yes	1
	No	0

SECTION 2: ANTENATAL CARE INFORMATION

READ OUT: In this section am going to be asking you about the health care that you received during your pregnancy.

**2.1**<br><br>Where was your pregnancy diagnosed/ confirmed?<br><br>Tick one block only	Public clinic/community health centre/MOU	1
	Public hospital	2
	Private doctor	3
	Private clinic/hospital	4
	Traditional healer	5
	Home pregnancy test	6
	Other (specify)	
**2.2**<br><br>Did you receive antenatal care services during your pregnancy?	Yes	1
	No	2
**2.3**<br><br>Where did your first antenatal visit for this pregnancy take place?	Public clinic/community health centre/MOU	1
	Public hospital	2
	Private doctor	3
	Private clinic/hospital	4
	Traditional healer	5

If no go to 2.6

Tick one block only	Other (specify)	
**2.4** How many months pregnant were you when you first received antenatal care?	_______________months	
**2.5** How many times did you attend the antenatal clinic during this pregnancy?	________________times	
**2.6** Were you offered an HIV test during this pregnancy?	Yes	1
	No	0
	Don’t know	99
**2.7** IF YES Did you agree to have the test?	Yes	1
	No	0
	Don’t know	99
**2.8** Were you told about the signs of pregnancy complications [warning signs]?	Yes	1
	No	0
	Don’t know	99
**2.9** IF YES Were you told where to go if you had these complications?	Yes	1
	No	0
	Don’t know	99

If no or don’t know go to 2.8

2.10 Apart from the care for your pregnancy, did you use any other health services during your pregnancy? Specify in relation to the calendar date	Type of facility or service	Yes	No	If yes, times used	If yes, amount spent
	Chemist/pharmacy	1	0		
	This clinic (not for	1	0		
	A different public	1	0		
	A private doctor	1	0		
	A traditional	1	0		
	A public hospital [illegible]	1	0		
	Inpatient stay in a	1	0		
	A private hospital [illegible]	1	0		
	Inpatient stay in a	1	0		
	ARV (HIV) clinic	1	0	Leave blank	
	TB clinic	1	0		

Read out each option one at a time. IF YES ask:<br><br>How many visits (or inpatient days) did you have?<br><br>Then ask:<br><br>How much did you have to pay the provider for each?<br><br>Tick all that apply "Yes" and others "No"	Other (Specify)	
2.11<br><br>Have you spent money on any other health care (excluding antenatal care) in the past month (e.g. traditional medicines, spaza shops, special food,	Yes	1
	No	0
	If Yes, specify amount<br><br>________________________________(Rand)	

etc). If YES, how much have you spent?		
2.12<br><br>Did you ever NOT use antenatal care health services when you needed them during your pregnancy?	Yes	1
	No	0

If no go to 2.14

2.13<br><br>IF YES<br><br>Why did you not use services?	
2.14<br><br>How many nights did you spend here for the birth?	________________number of nights

2.15<br><br>Did you have a normal birth or a caesar (caesarean section)?	Normal	1
	Caesarean	2
2.16<br><br>Is your baby alive or died at birth or shortly after?	Alive	1
	died	2
2.17<br><br>Is this your first delivery?	Yes	1
	No	0

If yes go to 2.19

2.18	

IF NO<br><br>How many deliveries have you had before?	

2.19<br><br>Have you ever had a baby die during pregnancy or birth?	Yes	1
	No	0

SECTION 3: ACCEPTABILITY

READ OUT: Can you tell me whether you agree or disagree with these statements when thinking about your general experience in this facility during labour and after birth

**3.1**<br><br>The doctors and nurses (*health workers)* explained what to expect when giving birth.	Agree	1
	Disagree	0
	Both agree and disagree	2
	Don't know / not sure	99
**3.2**<br><br>It is a problem that the *health workers* DO NOT speak my language.	Agree	1
	Disagree	0
	Both agree and disagree	2
	Don't know / not sure	99
**3.3**<br><br>The *health workers* understood the difficulty of being in labour and assisted me where possible.	Agree	1
	Disagree	0
	Both agree and disagree	2
	Don't know / not sure	99
**3.4**	Agree	1

The *health workers* were too busy to listen to my problems.	Disagree	0
	Both agree and disagree	2
	Don’t know / not sure	99
**3.5**<br><br>Some staff DO NOT treat patients with sufficient respect.	Agree	1
	Disagree	0
	Both agree and disagree	2
	Don’t know / not sure	99
**3.6**<br><br>The health workers I saw cared about me.	Agree	1
	Disagree	0
	Both agree and disagree	2
	Don’t know / not sure	99
**3.7**<br><br>I DID NOT receive sufficient pain relief during my labour	Agree	1
	Disagree	0
	Both agree and disagree	2
	Don’t know / not sure	99
**3.8**<br><br>The facilities (including waiting area and toilets) are dirty	Agree	1
	Disagree	0
	Both agree and disagree	2
	Don’t know / not sure	99
**3.9**<br><br>To what extent do you agree with the following statements about your pregnancy:<br><br>“I had all the support that I needed during my pregnancy from the father of the child”	Agree	1
	Disagree	2
	Don’t know	99
	Not applicable	98
**3.10**	Agree	1

"I had all the support that I needed from my family"	Disagree	2
	Don't know	99
	Not applicable	98
**3.11** "I had all the support that I needed from my friends"	Agree	1
	Disagree	2
	Don't know	99
	Not applicable	98
**3.12** In this facility are you able to talk to the doctors or nurses in private?	Always	1
	Sometimes	2
	Never	3

READ OUT: For the following questions, you are required to answer YES or NO

**3.13** Were you allowed to have a companion during your labour?	Yes	1 ← **If yes go to 3.15**
	No	0
	Don't know	99
**3.14** If no or don't know Would you have liked one?	Yes	1
	No	0
	Don't know	99
**3.15** Were you shouted at during labour?	Yes	1
	No	0
	Don't know	99
**3.16** Were you ever hit, slapped or pinched during labour?	Yes	1
	No	0
	Don't know	99

**3.17** Was your privacy respected?	Yes	1
	No	0
	Don't know	99
**3.18** Were you offered fluids?	Yes	1
	No	0
	Don't know	99
**3.19** Did you get referred for follow up care for you and the baby?	Yes	1
	No	0
	Don't know	99
**3.20** For birth registration, did you get all the necessary documents?	Yes	1
	No	0
	Don't know	99
**3.21** Were you told about the child-care grant & where to go for the child care grant if you qualify?	Yes	1
	No	0
	Don't know	99
**READ OUT:** For the last question, you are required to tell me how you were satisfied		
**3.22** How satisfied were you with the service you received during delivery?	Very satisfied/ Satisfied	1
	Neither satisfied nor dissatisfied	2
	Dissatisfied/ Very dissatisfied	3
	Don't know	99

Thank the interviewee and conclude interview !
