# Supplementary material for: A tool to define and measure maternal healthcare acceptability at a selected health sub-district in South Africa
Source: BMC Pregnancy Childbirth. 2023 Apr 29;23:302. doi: 10.1186/s12884-023-05475-y (PMC10148523; doi:10.1186/s12884-023-05475-y)
Supplement: Supplementary file 2 — Additional file 2: Table S1. Healthcare acceptability measurement tool using factor analysis. [file 12884_2023_5475_MOESM2_ESM.docx]

**Supplementary Table 1: Healthcare acceptability measurement tool using factor analysis**

| **HEALTHCARE ACCEPTABILITY MEASUREMENT TOOL**  **USING FACTOR ANALYSIS** | | | | | | | | |
| --- | --- | --- | --- | --- | --- | --- | --- | --- |
| Health Institution:  Service: Maternal healthcare  Data collection period: | | | | | | | | |
| **GENERAL CONDITIONS** | | | | | | | | |
|  | | Observed | | | Reference | | | |
| Number of included indicators for “Provider” construct | |  | | | Minimum 3 | | | |
| Number of included indicators for “Healthcare” construct | |  | | | Minimum 3 | | | |
| Number of included indicators for “Community” construct | |  | | | Minimum 3 | | | |
| Number of indicator response options (scale) | |  | | | Minimum 3 | | | |
| Number of participants (sample size): | |  | | | ≥ 250 | | | |
| **SUITABILITY** | | | | | | | | |
| Correlation matrix Bartlett’s test p-value | |  | | | < 0.05 | | | |
| Kaiser–Meyer–Olkin (KMO) measure of sampling adequacy | |  | | | > 0.50 | | | |
| **EXPLORATORY FACTOR ANALYSIS** | |  | | |  | | | |
| Number of retained factors* | |  | | | 3 | | | |
| Percentage of variability explained | |  | | | ≥ 0.60 | | | |
| **CONFIRMATION FACTOR ANALYSIS** | | | | | | | | |
| **Structural Equation Model (SEM) fitness** | | | | | | | | |
| chi-square p-value | |  | | | <0.05 | | | |
| Root mean square error of approximation (RMSA) | |  | | | <0.5 (ideal); (0.5-0.8): acceptable | | | |
| Comparative fit index (CFI) | |  | | | > 0.95 (ideal); (> 0.90): acceptable | | | |
| Tucker-Lewis index (TLI) | |  | | | > 0.95; (> 0.90): acceptable | | | |
| Standardized root mean residual (SRMR) | |  | | | <0.05 (ideal); (0.05-0.10): acceptable | | | |
| **Reliability** | |  | | |  | | | |
| Composite reliability (CR) | |  | | | > 0.70 (ideal);  0.45 – 0.70 (acceptable) | | | |
| Provider | |  | | |  |  |  |  |
| Healthcare | |  | | |  |  |  |  |
| Community | |  | | |  |  |  |  |
| Cronbach’s alpha value (Reliability) | |  | | | > 0.70 (ideal); (0.45- 70): acceptable | | | |
| Provider | |  | | |  |  |  |  |
| Healthcare | |  | | |  |  |  |  |
| Community | |  | | |  |  |  |  |
| **Validity** | |  | | |  | | | |
| Convergent validity (AVE) | |  | | | > 0.50 | | | |
| Provider | |  | | |  |  |  |  |
| Healthcare | |  | | |  |  |  |  |
| Community | |  | | |  |  |  |  |
| Discriminating validity | | AVE | | MSV | AVE > MSV | | | |
| Provider | |  | |  |  | | | |
| Healthcare | |  | |  |  | | | |
| Community | |  | |  |  | | | |
| **ACCEPTABILITY INDEX** | | | | | | | | |
| Scale range (1-100%) | | Mean | Std.dev | | Min | | Max | |
| Provider index | |  |  | |  | |  | |
| Healthcare Index | |  |  | |  | |  | |
| Community Index | |  |  | |  | |  | |
| Maternal healthcare index | |  |  | |  | |  | |
| **LIST OF INDICATORS INCLUDED**** | |  | | | | | | |
| **Provider construct variables** | **Healthcare construct variables** | **Community construct**  **variables** | | | | | | |
|  |  |  | | | | | | |
|  |  |  | | | | | | |
|  |  |  | | | | | | |
|  |  |  | | | | | | |
|  |  |  | | | | | | |
|  |  |  | | | | | | |
|  |  |  | | | | | | |
|  |  |  | | | | | | |
|  |  |  | | | | | | |
|  |  |  | | | | | | |
|  |  |  | | | | | | |
|  |  |  | | | | | | |
|  |  |  | | | | | | |
|  |  |  | | | | | | |
|  |  |  | | | | | | |
| **CONFIRMATION OF DATASET ATTACHED AS APPENDIX** | | | | | | Yes | |  |

***IF THE NUMBER OF RETAINED FACTORS DURING EXPLORATORY FACTOR ANALYSIS, IS DIFFERENT THAN 3 REPRESENTING PROVIDER, HEALTHCARE AND COMMUNITY RESPECTIVELY, THEN CONSIDER TO USE ARITHMETIC ANALYSIS METHOD TO CALCULATE MATERNAL HEALTHCARE ACCEPTABILITY**

**** IF MORE THAN 15 INDICATORS/VARIABLES HAVE BEEN INCLUDED, PLEASE WRITE DOWN EXTRAS ON THE BACK OF THE PAGE**
