# Supplementary material for: A tool to define and measure maternal healthcare acceptability at a selected health sub-district in South Africa
Source: BMC Pregnancy Childbirth. 2023 Apr 29;23:302. doi: 10.1186/s12884-023-05475-y (PMC10148523; doi:10.1186/s12884-023-05475-y)
Supplement: Supplementary file 3 — Additional file 3: Table S2. Healthcare acceptability measurement tool using simple arithmetic equation. [file 12884_2023_5475_MOESM3_ESM.docx]

**Supplementary Table 2: Healthcare acceptability measurement tool using simple arithmetic equation**

| **HEALTHCARE ACCEPTABILITY MEASUREMENT TOOL**  **USING SIMPLE ARITHMETIC ANALYSIS** | | | | | | |
| --- | --- | --- | --- | --- | --- | --- |
| Health Institution:  Service: Maternal healthcare  Data collection period: | | | | | | |
| **GENERAL CONDITIONS** | | | | | | |
|  | | Observed | | Reference | | |
| Number of included indicators for “Provider” construct | |  | | Minimum 3 | | |
| Number of included indicators for “Healthcare” construct | |  | | Minimum 3 | | |
| Number of included indicators for “Community” construct | |  | | Minimum 3 | | |
| Number of indicator response options (scale) | |  | | Minimum 3 | | |
| Number of participants (sample size) | |  | | ≥ 3 (nber of items x nber of scale) | | |
| **SUITABILITY** | |  | |  | | |
| Normalized indicators | |  | | Yes | | |
| Equal number of indicators per construct | |  | | Yes | | |
| **ACCEPTABILITY INDEX** | | | | | | |
| Scale range (1-100%) | | Mean | Std.dev | Min | Max | |
| Provider index | |  |  |  |  | |
| Healthcare Index | |  |  |  |  | |
| Community Index | |  |  |  |  | |
| Maternal healthcare index | |  |  |  |  | |
| **LIST OF INDICATORS INCLUDED*** | |  | | | | |
| **Provider construct variables** | **Healthcare construct variables** | **Community construct variables** | | | | |
|  |  |  | | | | |
|  |  |  | | | | |
|  |  |  | | | | |
|  |  |  | | | | |
|  |  |  | | | | |
|  |  |  | | | | |
|  |  |  | | | | |
|  |  |  | | | | |
|  |  |  | | | | |
|  |  |  | | | | |
|  |  |  | | | | |
|  |  |  | | | | |
|  |  |  | | | | |
|  |  |  | | | | |
|  |  |  | | | | |
| **CONFIRMATION OF DATASET ATTACHED AS APPENDIX** | | | | Yes | |  |

*** IF MORE THAN 15 INDICATORS/VARIABLES HAVE BEEN INCLUDED, PLEASE WRITE DOWN EXTRAS ON THE BACK OF THE PAGE**
